# Healthcare professionals’ queries on oseltamivir and influenza in Finland 2011‐2016—Can we detect influenza epidemics with specific online searches?

**DOI:** 10.1111/irv.12640

**Published:** 2019-03-06

**Authors:** Samuli Pesälä, Mikko J. Virtanen, Milla Mukka, Kimi Ylilammi, Pekka Mustonen, Minna Kaila, Otto Helve

**Affiliations:** ^1^ University of Helsinki Helsinki Finland; ^2^ National Institute for Health and Welfare Helsinki Finland; ^3^ Statomaly Ltd Helsinki Finland; ^4^ Duodecim Medical Publications Ltd Helsinki Finland; ^5^ Public Health Medicine University of Helsinki and Helsinki University Hospital Helsinki Finland; ^6^ Pediatric Research Center Children’s Hospital, University of Helsinki and Helsinki University Hospital Helsinki Finland

**Keywords:** health personnel, influenza, information systems, oseltamivir, primary health care, public health surveillance

## Abstract

**Background:**

Healthcare professionals (HCPs) search medical information during their clinical work using Internet sources. In Finland, Physician's Databases (PD) serve as an Internet medical portal aimed at HCPs. Influenza epidemics appear seasonal outbreaks causing public health concern. Oseltamivir can be used to treat influenza. Little is known about HCPs’ queries on oseltamivir and influenza from dedicated online medical portals and whether queries could be used as an additional source of information for disease surveillance when detecting influenza epidemics.

**Methods:**

We compared HCPs’ queries on oseltamivir and influenza from PD to influenza diagnoses from the primary healthcare register in Finland 2011‐2016. The Moving Epidemic Method (MEM) calculated the starts of influenza epidemics. Laboratory reports of influenza A and influenza B were assessed. Paired differences compared queries, diagnoses, and laboratory reports by using starting weeks. Kendall's correlation test assessed the season‐to‐season similarity.

**Results:**

We found that PD and the primary healthcare register showed visually similar patterns annually. Paired differences in the mean showed that influenza epidemics based on queries on oseltamivir started earlier than epidemics based on diagnoses by −0.80 weeks (95% CI: −1.0, 0.0) with high correlation (*τ* = 0.943). Queries on influenza preceded queries on oseltamivir by −0.80 weeks (95% CI: −1.2, 0.0) and diagnoses by −1.60 weeks (95% CI: −1.8, −1.0).

**Conclusions:**

HCPs’ queries on oseltamivir and influenza from Internet medical databases correlated with register diagnoses of influenza. Therefore, they should be considered as a supplementary source of information for disease surveillance when detecting influenza epidemics.

## 1 INTRODUCTION

Accessing the Internet enables users, including healthcare professionals (HCPs), to search medical information on the web. However, general search engines, such as Google, can lead to websites’ distributing medical information of varying quality to the general public, thus making it unreliable for use in clinical work. Online professional medical databases, such as MEDLINE or PubMed, provide information not only for scientific purposes but also for clinical decision making and continuing professional development.^1^ These databases are used to find information on current diseases and medications, and the information is used by HCPs in patient care.^2^, ^3^

Influenza appears worldwide as a possibly severe infectious disease with a major public health concern.^4^ Influenza outbreaks follow a temporal pattern and typically occur during the cold seasons of the year.^4^ People are infected mainly by two types of influenza viruses, A and B, spread via air or contaminated surfaces. Influenza epidemics may cause work absence, severe complications, hospitalizations, and even deaths, thus having a large economic burden on the community.^4^ For the prevention of influenza, immunization and good personal health and hygiene habits are suggested to control infection. To treat influenza, antiviral medications can be used. Oseltamivir, an antiviral agent, is used to treat seasonal or pandemic influenza in adults and children.^5^, ^6^ Oseltamivir is a neuraminidase inhibitor, which prevents the reproduction of the influenza virus. It is available both as a tablet and in a liquid form and is recommended for people with a high risk of complications and should be taken within 48 hours of the first symptoms of influenza.^4^, ^5^ The World Health Organization (WHO) has classified oseltamivir as complementary on their list of essential medicines in a health system.^7^

There are several methods for the detection of influenza epidemics. The number of influenza diagnoses has traditionally been collected during medical visits to primary healthcare professionals and also from positive findings at microbiological laboratories.^8^, ^9^ The accumulation of cases in a defined time period in a population determines the start of an influenza epidemic and indicates the intensity of an outbreak.^8^ Along with epidemiological and virological data from clinical encounters and microbiological laboratories, influenza search trends from Internet search engines have also been studied to detect epidemics.^10^, ^11^, ^12^, ^13^ Certain Google queries coincided highly with medical visits related to influenza‐like symptoms, thus making influenza activity estimation geographically possible.^13^ However, this surveillance method contained several flaws in timing and location of an influenza outbreak by overestimating the intensity of an epidemic and missing the first wave of an influenza pandemic.^14^ In addition, searches of the general public may be affected by issues not related to an actual epidemic, such as publicity on a given disease while searches by HCPs may be less affected.^15^ As general search engines are used by both the general public and HCPs, these search data can yield heterogeneous results. Our study on HCPs’ information seeking behavior from Internet medical databases showed that searches and diagnoses on Lyme borreliosis associated with each other.^16^ Therefore, we concluded that Internet searches could be used as an additional source of information for disease surveillance.^16^

The Moving Epidemic Method (MEM) has been developed to assess the timing of influenza epidemics and to estimate their baseline and threshold.^17^ It is implemented by WHO and the European Centres for Disease Prevention and Control (ECDC) to monitor influenza circulation in European countries.^17^, ^18^, ^19^ Historical data on influenza weekly rates are analyzed with MEM, and the method includes three stages.^17^, ^18^, ^19^ In the first stage, the length of each influenza season with start and end points is determined forming a pre‐epidemic, an epidemic, and a post‐epidemic period. In the second stage, the epidemic baseline and thresholds are calculated using pre‐epidemic and post‐epidemic values from historical seasons. In the third stage, low, medium, and high intensity thresholds are computed. Although early detection of influenza to detect outbreaks using epidemiological and virological data has been studied before, including general search engine queries, little is known about queries on influenza and oseltamivir on Internet databases by HCPs.

When searching for medical information on influenza and oseltamivir, HCPs access dedicated medical databases on the Internet. Duodecim Medical Publications Ltd (owned by the Finnish Medical Society Duodecim) produces and maintains an Internet‐based portal called Physician's Databases (PD).^20^ It is available throughout the Finnish healthcare system, and its users can be tracked in primary health care in Finland by an Internet Protocol address included in a log file. PD includes point‐of‐care evidence‐based medicine articles aimed at HCPs, mostly comprising physicians, pharmacists, and nurses. In 2016, there were nearly 21 000 working‐age physicians in Finland.^21^ The medical articles in the databases are in Finnish and are opened over 15 million times a year. The National Institute for Health and Welfare (NIHW) is the research and development institute in Finland maintaining the register of public primary healthcare diagnoses and the National Infectious Diseases Register (NIDR).^22^ The data from both NIHW registers can be used in research, decision making, and planning of healthcare services.

When searching for medical information on influenza in clinical work, primary care physicians in Finland may access the medical Internet portal, PD, where a medical article on influenza aimed at HCPs can be found. During or immediately following a patient visit, a primary care physician reports an influenza diagnosis in the electronic patient record, where it will then be automatically transferred to the national register of public primary healthcare diagnoses. NIDR includes laboratory reports of influenza A and influenza B notified electronically by microbiological laboratories. If the prescription of an antiviral medication for influenza is needed, PD's pharmaceutical database is available for a physician to search for information on oseltamivir. Queries on oseltamivir (openings of the page with information on oseltamivir) and influenza (openings of the page with information on influenza) during a physician's encounter can be tracked in the log files of PD. The aim of our study was to assess whether HCPs’ queries on oseltamivir and influenza from Internet medical databases could be used as an additional source of information for disease surveillance when detecting influenza epidemics. We hypothesized that queries on oseltamivir would share similar seasonal trends seen in epidemiological data on influenza and that they could be measured with MEM.

## 2 MATERIAL AND METHODS

We carried out a register‐based study by collecting weekly log data on the number of queries on oseltamivir and influenza from PD, in order to compare logs to influenza diagnoses (J09‐11 according to the *International Statistical Classification of Diseases and Related Health Problems*, 10th Revision [ICD‐10] disease classification code system^9^ and R80 in the *International Classification of Primary Care, Second Edition* [ICPC2] coding system^23^) and laboratory reports of influenza A and influenza B found from NIDR. Queries on oseltamivir included log data on oral capsules (30 mg, 45 mg, and 75 mg) and a powder for oral suspension (6 mg/mL) of oseltamivir. The data were collected across Finland during 2011‐2016 comprising five seasons of influenza (2011/12, 2012/13, 2013/14, 2014/15, and 2015/16) with five indicators (queries on oseltamivir, influenza diagnoses, laboratory reports of influenza A and influenza B, and queries on influenza).

We used the MEM model to calculate the starts and ends of an epidemic period and influenza thresholds (pre‐epidemic, post‐epidemic) [R language, 2.12 version^24^]. We analyzed the starting weeks of the epidemic periods consisting of queries on oseltamivir, influenza diagnoses, queries on influenza, and laboratory reports of influenza A and B pairwise comprising a total of ten pairs. The starting weeks correspond to week numbers for a calendar year starting from the beginning of January (week 1). To assess if each indicator reaches the epidemic threshold at similar times, paired differences in the starting weeks were calculated. Due to a small number of observations (starting weeks), the bootstrapping method^25^, ^26^ was used to estimate the distribution of observations. We bootstrapped paired differences comprising five observations with 1,000 replications resulting in bootstrapped mean, bias‐corrected and accelerated (BCa) (adjusted for ties) 95% confidence interval (CI) of the mean, and p‐value of the mean. Kendall's rank correlation coefficient (*τ*) was used to assess the statistical season‐to‐season similarity between a pair (Kendall's tau formula in Appendix 1 files). Paired differences and Kendall's correlation tested only the starting weeks comprising five seasons with five indicators.

## 3 RESULTS

**Figure 1:**
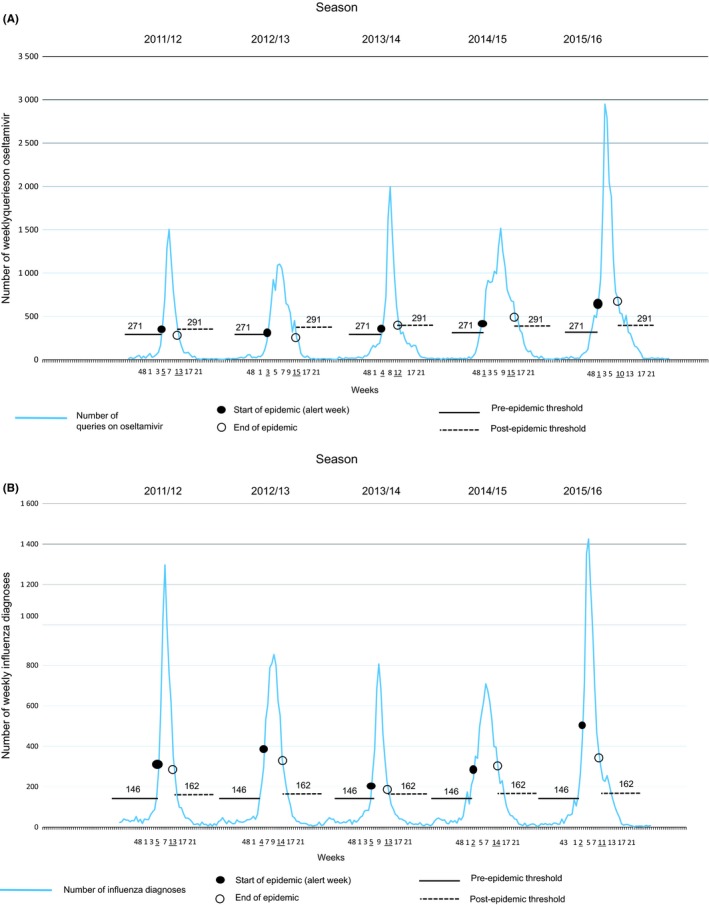
Influenza epidemic thresholds (pre‐epidemic, post‐epidemic) across Finland during 2011‐2016 by season for (A) weekly queries on oseltamivir and (B) weekly influenza diagnoses. The start and end of an epidemic period placed in the patterns of queries and diagnoses corresponds to the underlined epidemic week

**Figure 2:**
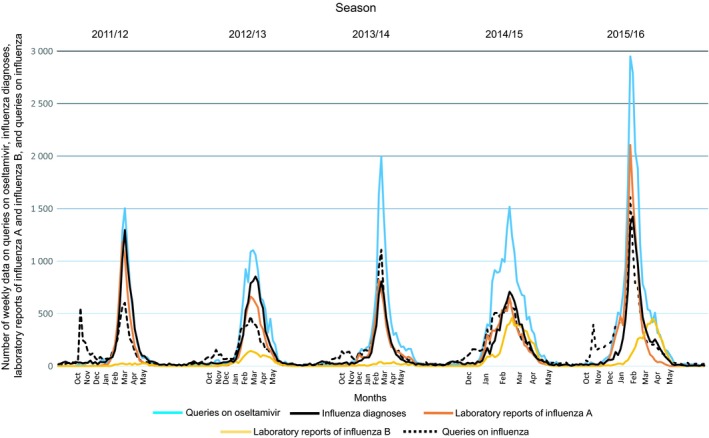
Queries on oseltamivir, influenza diagnoses, laboratory reports of influenza A and influenza B, and queries on influenza across Finland during 2011‐2016 by season

**Figure 3:**
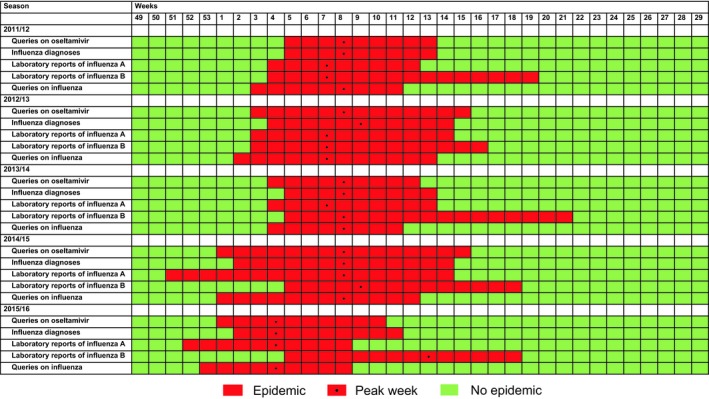
The MEM‐calculated epidemic weeks (red) and non‐epidemic weeks (green) on queries on oseltamivir, influenza diagnoses, laboratory reports of influenza A and influenza B, and queries on influenza by season. The black bullets indicate peak weeks during epidemic periods

Visually similar patterns were found between annual queries on oseltamivir and influenza diagnoses during 2011‐2016 by season (Figure 1, panel A and B). In addition, laboratory reports of influenza A and influenza B and queries on influenza shared similar seasonal patterns (Figure 2). The MEM‐calculated weekly queries on oseltamivir start during weeks 1‐5 (alert weeks) and end during weeks 10‐15 (Figure 1, panel A and Table 1). The seasons peak during weeks 4‐8 (Figure 3). Pre‐ and post‐epidemic thresholds throughout the seasons were at 271 and 291 queries on oseltamivir, respectively. Influenza diagnoses calculated by MEM start during weeks 2‐5 (alert weeks) and end during weeks 11‐14 (Figure 1, panel B and Table 1). The seasons peak during weeks 4‐9 (Figure 3). Pre‐ and post‐epidemic thresholds throughout the seasons were at 146 and 162 influenza diagnoses, respectively. Table 1 and Figure 3 show the MEM‐calculated starts and ends of the epidemic periods. Paired differences were computed by using the starting weeks. Paired differences in the mean showed statistical significance between queries on oseltamivir and influenza diagnoses (−0.80 weeks, 95% BCa CI: −1.0, 0.0, *P* = 0.000) and between diagnoses and laboratory reports of influenza A (2.00 weeks, 95% BCa CI: 1.0, 3.2, *P* = 0.000). Very high positive correlations were found between queries on oseltamivir and influenza diagnoses (*τ* = 0.943) and between diagnoses and laboratory reports of influenza A (*τ* = 0.943). Queries on influenza preceded queries on oseltamivir by −0.80 weeks (95% BCa CI: −1.2, 0.0, *P* = 0.015), diagnoses by −1.60 weeks (95% BCa CI: −1.8, −1.0, *P* = 0.000), and laboratory reports of influenza A by −0.80 weeks (95% BCa CI: −1.8, 0.4, *P* = 0.166) and B by −2.40 weeks (95% BCa CI: −3.8, −1.0, *P* = 0.002) (Figure 2, Table 2). In addition, paired differences in the mean showed statistical significance between queries on oseltamivir and laboratory reports of influenza A (1.20 weeks, 95% BCa CI: 0.0, 2.0, *P* = 0.021). Very high positive correlation appeared between queries on influenza and influenza diagnoses (*τ* = 0.894) and queries on oseltamivir and laboratory reports of influenza A (*τ* = 0.889). Indicators paired with laboratory reports of influenza B showed negative correlations. However, statistically significant difference was found when laboratory reports of influenza A were paired with laboratory reports of influenza B (−2.80 weeks, 95% BCa CI: −5.6, −0.4, *P* = 0.041, *τ* = −0.252). The results of the paired differences in the mean, BCa CIs, p‐values, and correlations are shown in Table 2.

**Table 1:** The MEM‐calculated starts and ends of the epidemic periods on queries on oseltamivir, influenza diagnoses, laboratory reports of influenza A and influenza B, and queries on influenza across Finland by season

Season	Queries on oseltamivir		Influenza diagnoses		Laboratory reports of influenza A		Laboratory reports of influenza B		Queries on influenza	
	Epidemic starts		Epidemic starts		Epidemic starts		Epidemic starts		Epidemic starts	
	Date	Week	Date	Week	Date	Week	Date	Week	Date	Week
Start of epidemics										
2011/12	Jan 30	5	Jan 30	5	Jan 23	4	Jan 23	4	Jan 16	3
2012/13	Jan 14	3	Jan 21	4	Jan 14	3	Jan 14	3	Jan 7	2
2013/14	Jan 20	4	Jan 27	5	Jan 20	4	Jan 27	5	Jan 20	4
2014/15	Dec 29	1	Jan 5	2	Dec 15	51	Jan 26	5	Dec 29	1
2015/16	Jan 4	1	Jan 11	2	Dec 21	52	Feb 1	5	Dec 28	53

Season	Queries on oseltamivir		Influenza diagnoses		Laboratory reports of influenza A		Laboratory reports of influenza B		Queries on influenza	
	Epidemic ends		Epidemic ends		Epidemic ends		Epidemic ends		Epidemic ends	
	Date	Week	Date	Week	Date	Week	Date	Week	Date	Week
End of epidemics										
2011/12	Mar 26	13	Mar 26	13	Mar 19	12	May 7	19	Mar 12	11
2012/13	Apr 8	15	Apr 1	14	Apr 1	14	Apr 15	16	Mar 25	13
2013/14	Mar 17	12	Mar 24	13	Mar 24	13	May 19	21	Mar 10	11
2014/15	Apr 6	15	Mar 30	14	Mar 30	14	Apr 27	18	Mar 16	12
2015/16	Mar 7	10	Mar 14	11	Feb 22	8	May 2	18	Feb 22	8

**Table 2:** Pairs, paired differences with the mean, bias‐corrected and accelerated confidence intervals and p‐values, and Kendall's correlations. Queries on oseltamivir, influenza diagnoses, laboratory reports of influenza A and influenza B, and queries on influenza were paired and calculated and bootstrapped according to the start of the epidemic weeks shown in Table 1

Pair	Paired differences				Kendall's correlation coefficient (*τ*)
	Mean	Bias‐corrected and accelerated 95% confidence interval of the mean (adjusted for ties)		*p*‐value of the mean	
		Lower	Upper		
Queries on oseltamivir – Influenza diagnoses	−0.80	−1.0	0.0	0.000	0.943
Influenza diagnoses – Laboratory reports of influenza A	2.00	1.0	3.2	0.000	0.943
Queries on influenza – Influenza diagnoses	−1.60	−1.8	−1.0	0.000	0.894
Queries on oseltamivir – Laboratory reports of influenza A	1.20	0.0	2.0	0.021	0.889
Queries on influenza – Queries on oseltamivir	−0.80	−1.2	0.0	0.015	0.738
Queries on influenza – Laboratory reports of influenza A	−0.80	−1.8	0.4	0.166	0.738
Queries on influenza – Laboratory reports of influenza B	−2.40	−3.8	−1.0	0.002	−0.120
Laboratory reports of influenza A – of influenza B	−2.80	−5.6	−0.4	0.041	−0.252
Influenza diagnoses – Laboratory reports of influenza B	−0.80	−2.4	1.0	0.354	−0.267
Queries on oseltamivir – Laboratory reports of influenza B	−1.60	−3.2	0.4	0.088	−0.378

## 4 DISCUSSION

Our study showed similar patterns and statistically significant paired differences and high correlations between HCPs’ queries on oseltamivir and primary healthcare influenza diagnoses during the starting weeks of an epidemic in Finland during 2011‐2016 (Figure 1, panel A and B, Table 2). In addition, high correlations and statistically significant paired differences were found between queries on oseltamivir and laboratory reports of influenza A, queries on influenza and oseltamivir, and queries on influenza and influenza diagnoses (Table 2). Paired differences estimated how much earlier the epidemic started. The smaller the p‐value, the more statistically significant the paired difference was. The higher the correlation, the more similarly a paired indicator appeared between seasons. Pairs with negative correlations related to laboratory reports of influenza B (Table 2) indicate that the epidemics of influenza B appear seasonally later than influenza A (Figures 2 and 3).

Using the Internet enables users, including HCPs, to search for health‐related information on diseases and medications.^2^, ^3^ Internet platforms may provide HCPs with medical information of questionable quality.^27^ Although influenza searches from Google have been assessed in terms of disease surveillance, this method included several weaknesses in timing and regional scales to predict influenza epidemics.^12^, ^13^, ^14^ Notably, general search engines cannot characterize the users performing the searches. We have shown here that HCPs’ queries on oseltamivir and influenza from the dedicated Internet portal highly coincided with diagnoses and laboratory reports of influenza A and influenza B.

This study includes certain limitations to be taken into consideration. In this work, we studied queries on oseltamivir and influenza in the whole country including no data on geographical variations. The influenza epidemic may start in different regions of a country at different times, and the detection of this would require more accurate location data. PD and the national register of public primary healthcare diagnoses are separate databases; thus, queries and diagnoses cannot be connected to one another nor the same patient visited primary care physician's encounter. It is possible that the great number of queries on oseltamivir (Figure, panel A) compared to influenza diagnoses (Figure, panel B) also includes the presence of some secondary healthcare queries on oseltamivir opened from PD. In addition, during influenza epidemics, the national public primary healthcare register may exclude some influenza diagnoses that were misdiagnosed and reported as within a broader category of infectious diseases, such as acute respiratory infections. Also, not every patient presenting influenza symptoms is tested for the virus. In these cases, the prescription is based on symptoms and knowledge on the epidemic situation of the influenza virus in the given population. In the Finnish primary health care, oseltamivir is rarely prescribed outside influenza epidemic periods and is, therefore, less well‐known than medications that are constantly prescribed. This results in queries on oseltamivir specifically during epidemics. However, when treating influenza patients, physicians may still only check up on indications of oseltamivir from PD without then prescribing the medication, thus increasing the number of queries in a log file. Also, media coverage may affect HCPs’ queries during and outside epidemics.^15^, ^28^ Not only influenza publications in the media may impact HCPs themselves, but also patients may face influenza‐related media publications. During clinical encounters, patients may present certain symptoms or demand influenza medication to stockpile them worrying pandemics,^29^ resulting in possible search trends from PD. Since queries on influenza from PD preceded other indicators (Table 1, Figures 2 and 3), HCPs may have searched influenza‐related information during encounters prior to an epidemic. In addition, the magnitudes between the patterns of queries on oseltamivir and diagnoses may also vary due to different qualities of the databases. However, since MEM was used to calculate only starts and ends of the epidemic periods, the heights of the spikes did not have an effect on the outcomes of the analyses. The strength of our study was that HCPs (representativeness) search for information from real‐time Internet databases (timeliness).

This is the first study, to our knowledge, to demonstrate high correlation and statistical significance between queries on oseltamivir and primary healthcare influenza diagnoses by using the MEM model. We found that HCPs’ information searching behavior strongly associates with epidemiological data on influenza. Therefore, we state that HCPs’ queries could be used as a supplementary source of information for disease surveillance when detecting influenza epidemics. Our study depicts a possible development for infectious disease surveillance systems. While our study utilizes a database unique for Finland, similar medical databases can be used to assess data in European countries and internationally.^9^, ^19^ The combination of Internet‐based query data and other surveillance data could enhance current surveillance systems. In the future, it may be possible to create algorithms that analyze HCPs’ queries in real time in order to help the detection of the beginning epidemic. Information from these different sources could be combined and delivered to primary healthcare units facing the first patients at the start of an epidemic to estimate the need for primary healthcare services and workforce during epidemics. Further studies should focus on the applicability of these results in different pathologies and other medical databases in other countries.

## CONFLICT OF INTERESTS

MK has held various trustee positions in the Finnish Medical Society Duodecim since the late 1990s. OH has held various trustee positions in the Finnish Medical Society Duodecim and Duodecim Medical Publications Ltd since 2009 and is a partner at iHealth Finland Ltd. The other authors have no competing interests.

## AUTHORS’ CONTRIBUTIONS

SP, MJV, MM, KY, PM, MK, and OH designed the study concept. SP, MJV, MM, and OH performed the literature research. SP, MJV, KY, and OH gathered and supplied the study data. SP, MJV, and KY carried out the data analysis. SP, MJV, and OH carried out the data interpretation. SP, MJV, MM, KY, PM, MK, and OH involved in the critical revision and final approval. SP and MM drafted the manuscript.

## APPENDIX 1

Kendall's tau formula.

## References

[1] Younger P . Internet‐based information‐seeking behaviour amongst doctors and nurses: a short review of the literature. Health Info Libr J. 2010;27(1):2‐10. 20402799 10.1111/j.1471-1842.2010.00883.x

[2] Del Fiol G , Workman TE , Gorman PN . Clinical questions raised by clinicians at the point of care: a systematic review. JAMA Intern Med. 2014;174(5):710‐718. 24663331 10.1001/jamainternmed.2014.368

[3] Clarke MA , Belden JL , Koopman RJ , et al. Information needs and information‐seeking behaviour analysis of primary care physicians and nurses: a literature review. Health Info Libr J. 2013;30(3):178‐190. 23981019 10.1111/hir.12036

[4] Factsheet about seasonal influenza (ECDC). Available from: https://ecdc.europa.eu/en/seasonal-influenza/facts/factsheet. Accessed November 28, 2018.

[5] Dobson J , Whitley RJ , Pocock S , Monto AS . Oseltamivir treatment for influenza in adults: a meta‐analysis of randomised controlled trials. Lancet. 2015;385(9979):1729‐1737. 25640810 10.1016/S0140-6736(14)62449-1

[6] Malosh RE , Martin ET , Heikkinen T , Brooks WA , Whitley RJ , Monto AS . Efficacy and safety of oseltamivir in children: Systematic review and individual patient data meta‐analysis of randomized controlled trials. Clin Infect Dis. 2018;66(10):1492‐1500. 29186364 10.1093/cid/cix1040

[7] World Health Organization . Model List of Essential Medicines 20th List. March 2017. Available from: http://www.who.int/medicines/publications/essentialmedicines/20th_EML2017.pdf. Accessed November 28, 2018.

[8] Cooper DL , Verlander NQ , Elliot AJ , Joseph CA , Smith GE . Can syndromic thresholds provide early warning of national influenza outbreaks? J Public Health (Oxf). 2009;31(1):17‐25. 18032426 10.1093/pubmed/fdm068

[9] World Health Organization . Global Epidemiological Surveillance Standards for Influenza (July 2012). ISBN: 978924 1506601. Available from: http://www.who.int/influenza/resources/documents/WHO_Epidemiological_Influenza_Surveillance_Standards_2014.pdf?ua=1 Accessed November 28, 2018.

[10] Polgreen PM , Chen Y , Pennock DM , Nelson FD . Using internet searches for influenza surveillance. Clin Infect Dis. 2008;47(11):1443‐1448. 18954267 10.1086/593098

[11] Yuan Q , Nsoesie EO , Lv B , Peng G , Chunara R , Brownstein JS . Monitoring influenza epidemics in China with search query from Baidu. PLoS ONE. 2013;8(5):e64323. 23750192 10.1371/journal.pone.0064323 PMC3667820

[12] Infodemiology EG . tracking flu‐related searches on the web for syndromic surveillance. AMIA Ann Symp Proc. 2006;2006:244‐248. PMC1839505 17238340

[13] Ginsberg J , Mohebbi MH , Patel RS , Brammer L , Smolinski MS , Brilliant L . Detecting influenza epidemics using search engine query data. Nature. 2009;457(7232):1012‐1014. 19020500 10.1038/nature07634

[14] Olson DR , Konty KJ , Paladini M , Viboud C , Simonsen L . Reassessing Google Flu Trends data for detection of seasonal and pandemic influenza: a comparative epidemiological study at three geographic scales. PLoS Comput Biol. 2013;9(10):e1003256. 24146603 10.1371/journal.pcbi.1003256 PMC3798275

[15] Pesälä S , Virtanen MJ , Sane J , Mustonen P , Kaila M , Helve O . Health information–seeking patterns of the general public and indications for disease surveillance: register‐based study using Lyme disease. JMIR Public Health Surveill. 2017;3(4):e86. 29109071 10.2196/publichealth.8306 PMC5696583

[16] Pesälä S , Virtanen MJ , Sane J , et al. Health care professionals' evidence‐based medicine Internet searches closely mimic the known seasonal variation of Lyme borreliosis: a register‐based study. JMIR Public Health Surveill. 2017;3(2):e19. 28400357 10.2196/publichealth.6764 PMC5405287

[17] Vega T , Lozano JE , Meerhoff T , et al. Influenza surveillance in Europe: establishing epidemic thresholds by the moving epidemic method. Influenza Other Respir Viruses. 2013;7(4):546‐558. 22897919 10.1111/j.1750-2659.2012.00422.x PMC5855152

[18] Vega T , Lozano JE , Meerhoff T , et al. Influenza surveillance in Europe: comparing intensity levels calculated using the moving epidemic method. Influenza Other Respir Viruses. 2015;9(5):234‐246. 26031655 10.1111/irv.12330 PMC4548993

[19] Murray J , Marques D , Cameron RL , et al. Moving epidemic method (MEM) applied to virology data as a novel real time tool to predict peak in seasonal influenza healthcare utilisation. The Scottish experience of the 2017/18 season to date. Euro Surveill. 2018;23(11);18‐00079. 10.2807/1560-7917.ES.2018.23.11.18-00079 PMC5861591 29560854

[20] Duodecim Medical Publications Ltd . Available from: http://www.duodecim.fi/english Accessed December 4, 2018.

[21] The Finnish Medical Association: Physicians 2016. Available from: http://www.laakariliitto.fi/site/assets/files/1011/ll16_taskutil_06_en_160524net.pdf Accessed November 28, 2018 .

[22] Infectious diseases in Finland. 2016. Available from http://urn.fi/URN:ISBN:978‐952‐302‐978‐1 Accessed November 28, 2018

[23] International Classification of Primary Care, Second edition (ICPC‐2). Available from: http://www.who.int/classifications/icd/adaptations/icpc2/en/ Accessed November 28, 2018.

[24] Lozano Alonso JE . Mem: Moving Epidemics Method, R Package, version 2.12;2018. Available from: https://cran.r-project.org/web/packages/mem/mem.pdf Accessed December 4, 2018.

[25] Boianelli A , Nguyen VK , Ebensen T , et al. Modelling Influenza Virus Infection: A Roadmap for Influenza Research. Viruses. 2015;7(10):5274‐5304. 26473911 10.3390/v7102875 PMC4632383

[26] Bootstrapped Confidence Intervals for the Mean and the Median: SPSS. Karl L. Wuensch, March 2018. East Carolina University, 2018. Available from: http://core.ecu.edu/psyc/wuenschk/SPSS/Bootstrap_SPSS.pdf Accessed December 12, 2018.

[27] Chou WY , Prestin A , Lyons C , Wen KY . Web 2.0 for health promotion: reviewing the current evidence. Am J Public Health. 2013;103(1):e9‐18. 10.2105/AJPH.2012.301071 PMC3518341 23153164

[28] Kostkova P , Fowler D , Wiseman S , Weinberg JR . Major infection events over 5 years: how is media coverage influencing online information needs of health care professionals and the public? J Med Internet Res. 2013;15(7):e107. 23856364 10.2196/jmir.2146 PMC3713905

[29] Centers for Disease Control and Prevention (CDC) . Increased antiviral medication sales before the 2005–06 influenza season–New York City. MMWR Morb Mortal Wkly Rep. 2006;55(10):277‐279. 16543882

